# Physiological and transcriptional responses of lactic acid bacteria to sub-inhibitory cadmium exposure

**DOI:** 10.3389/fmicb.2026.1914052

**Published:** 2026-10-13

**Authors:** Diana Vardanyan, Leonardo Mancini, Matteo Bernardi, Pasquale Filannino, Francesco Maria Calabrese, Maria De Angelis

**Affiliations:** Department of Soil, Plant and Food Science, University of Bari Aldo Moro, Bari, Italy

**Keywords:** cadmium stress, gene regulation, heavy metal resistance, lactic acid bacteria, membrane lipid remodeling, physiological adaptation

## Abstract

**Introduction:**

Cadmium is a widespread environmental contaminant that can adversely impact microbial growth and metabolism. While the ability of Lactic acid bacteria (LAB) to bind and sequester cadmium has been extensively investigated, less is known about their physiological adaptation to sub-inhibitory cadmium exposure.

**Methods:**

Following the initial determination of the minimum inhibitory concentration (MIC), four selected LAB strains were exposed to sub-inhibitory cadmium concentrations (1/8, 1/4 and 1/2 × their respective MIC values) and investigated using a combined physiological and transcriptional approach. Growth performance, culturability, viability, and acidification activity were assessed alongside the expression of *cadA, cadD, mntH*, and *czcA*, genes involved in metal transport, homeostasis, and detoxification, respectively.

**Results:**

The results revealed marked strain-dependent differences in cadmium tolerance and adaptation. Even at sub-inhibitory concentrations, cadmium affected bacterial physiology by reducing growth and acidification activity. *Limosilactobacillus reuteri* showed the strongest reduction in relative growth, which decreased to 18.8% of the untreated control at $\frac{1}{2}$ × MIC, while Nile Red fluorescence increased up to 5.61-fold, indicating pronounced alterations in the membrane lipid environment. LIVE/DEAD analyses demonstrated a concentration-dependent increase in the population of membrane-compromised cells, although proportional losses in cell viability did not always accompany reductions in culturability. Nile Red fluorescence analyses further revealed significant alterations in the cellular lipid environment, suggesting membrane remodeling as an important component of the adaptive response to cadmium exposure. Transcriptional analyses further showed heterogeneous expression patterns among strains, suggesting that adaptation to cadmium is regulated through strain-specific responses rather than a shared regulatory strategy.

**Discussion:**

Overall, these findings indicate that LAB responses to cadmium involve strain-dependent physiological and transcriptional changes. The study highlights the importance of considering strain-specific traits when evaluating bacterial responses to metal stress and provides a basis for future investigations into the molecular mechanisms underlying adaptation to sub-lethal cadmium exposure.

## 1 Introduction

Cadmium (Cd) is among the most harmful heavy metals due to its high toxicity, persistence, and propensity to bioaccumulate (Silver and Phung, 2005; Khan et al., 2022; Davidova et al., 2024). Even at low concentrations, cadmium presents a substantial risk to environmental and human health (Edo et al., 2024; Guo et al., 2025; Shobowale et al., 2025; Vijiyakumar and Prince, 2025). It disrupts divalent metal-ion homeostasis by perturbing trace cation transport networks (e.g., MntH and ZnuABC) and suppresses central bioenergetic pathways, specifically the Embden–Meyerhof–Parnas (glycolytic) route, pyruvate fermentation, and F_1_F_0_-ATP synthase-driven proton motive force maintenance. Concurrently, it can perturb thioredoxin/glutathione redox networks and molecular chaperone systems (such as DnaK/GroEL), inducing acute oxidative stress, cell membrane compromise, and growth arrest (Farahat et al., 2025; Mazumder et al., 2026; Sugiyono et al., 2026).

Lactic acid bacteria (LAB) are Gram-positive, facultative anaerobic microorganisms widely distributed in nature and encompassing a wide range of species. Generally regarded as safe (GRAS), LAB play an important role in the human gut microbiota and are extensively used in food fermentation processes, including dairy and bakery products, and functional foods (Jadán-Piedra et al., 2017; De Vuyst and Leroy, 2020; Beglari et al., 2023; Vanitha et al., 2023).

In recent years, increasing evidence has been presented for the ability of LAB to interact with heavy metals, including cadmium, due to its reduced bioavailability in environmental and host-associated systems (Ma, 2024). This interaction is mediated by multiple processes, including surface adsorption, intracellular accumulation and detoxification mechanisms (Li et al., 2021; Petrova et al., 2022; Beheshtian et al., 2024; Ma, 2024). Firstly, cadmium ions can be adsorbed on the cell surface by negatively charged components, including peptidoglycan, teichoic acids, surface proteins, and extracellular polysaccharides (Chapot-Chartier and Kulakauskas, 2014). Functional groups such as carboxyl, hydroxyl, and sulfhydryl moieties provide binding sites for metal ions, promoting their immobilization and reducing their immediate bioavailability (Yang et al., 2023). This passive process is considered one of the main mechanisms underlying the bioremediation potential of LAB (Ma, 2024).

Once cadmium enters the cell, bacterial adaptation relies on a range of intracellular mechanisms aimed at limiting metal accumulation and toxicity (Nnaji et al., 2024; Adhikary et al., 2024). These include active efflux systems, such as P-type ATPases (*cadA*) and resistance-nodulation-division (*RND*) transporters (*czcA*), which reduce intracellular cadmium concentrations through energy-dependent export processes. In parallel, transporters involved in metal homeostasis, including members of the NRAMP family such as MntH, may indirectly contribute to cadmium uptake due to the chemical similarity between Cd^2^^+^ and essential divalent cations (Patzer and Hantke, 2001; Forbes and Gros, 2001; Sasaki et al., 2012). Additional tolerance determinants, including accessory proteins such as *cadD* and metal-binding intracellular components, further contribute to mitigating metal-induced cellular damage (Tsai et al., 1992; Lee et al., 2001; Chandrangsu et al., 2017; Ducret et al., 2020).

Besides metal transport and detoxification systems, maintenance of membrane integrity represents a critical component of bacterial adaptation to heavy-metal stress. Exposure to toxic metals has been shown to alter membrane fluidity, phospholipid organization, fatty acid composition, and membrane permeability, thereby affecting cellular homeostasis and survival (Zhang and Rock, 2008; Siliakus et al., 2017). Membrane remodeling is increasingly recognized as an important adaptive response that enables bacteria to preserve membrane functionality under adverse environmental conditions. However, despite the growing interest in LAB-mediated cadmium removal, little information is available regarding membrane-associated responses and lipid reorganization in LAB exposed to cadmium stress.

As a consequence, cadmium tolerance in LAB cannot be attributed to a single resistance determinant, but rather emerges from the coordinated action of multiple physiological and molecular processes regulating metal uptake, sequestration, detoxification, and efflux (Ilusanya et al., 2024; Abbas et al., 2025; Oleńska et al., 2025). The efficiency of these systems ultimately determines intracellular cadmium retention and cellular adaptation under metal stress conditions (Nies, 2003; Seiler and Berendonk, 2012). However, maintaining metal homeostasis is energetically demanding, since active transport, stress response, and repair mechanisms require substantial cellular resources, potentially affecting growth and metabolic performance under prolonged or intense cadmium exposure (Nies, 2003; Sharma et al., 2024).

While the contribution of LAB to cadmium removal through adsorption and sequestration mechanisms has been extensively investigated, considerably less attention has been dedicated to understanding how LAB cells physiologically adapt to cadmium exposure and regulate intracellular metal homeostasis under sub-inhibitory stress conditions (Djukić-Vuković et al., 2022). In fact, under stress, sub-inhibitory cadmium concentrations were selected because they impose physiologically relevant metal stress while maintaining bacterial growth and metabolic activity, thereby enabling the investigation of adaptive regulatory, homeostatic, and membrane-associated responses that are often obscured under fully inhibitory conditions (Nies, 2003; Chandrangsu et al., 2017). In this study, we employed a combined physiological and transcriptional approach to explore the underlying mechanisms of cadmium exposure under sub-inhibitory stress conditions in selected LAB strains. In detail, we evaluated the effects of cadmium on bacterial growth, cultivability, viability, acidification activity and intracellular lipid environment, while assessing the expression of selected genes *cadA, cadD, mntH*, and *czcA* based on their representative transcriptional marker activity, covering complementary adaptive processes involved in cadmium extrusion, intracellular metal homeostasis, and heavy-metal detoxification. By integrating phenotypic and transcriptional responses, we aimed at highlighting strain-specific regulatory approaches employed by LAB to tolerate cadmium stress.

## 2 Materials and methods

### 2.1 Bacterial strains, culture conditions and determination of minimum inhibitory concentration (MIC)

A total of eleven LAB strains, namely *Lactiplantibacillus plantarum* DSM 20174 (*Lp. plantarum*), *Fructilactobacillus rossiae* DSM 15814 (*Fr. rossiae*), *Latilactobacillus sakei* DSM 20017 (*Lt. sakei*), *Weissella confusa* DSM 20196 (*W. confusa*), *Weissella cibaria* DSM 14295 (*W. cibaria*), *Leuconostoc citreum* DSM 5577 (*L. citreum*), *Pediococcus pentosaceus* DSM 20336 (*P. pentosaceus*), *Lactococcus lactis* DSM 20481 (*Lc. lactis*), *Latilactobacillus curvatus* DSM 20019 (*Lt. curvatus*), *Fructilactobacillus sanfranciscensis* DSM 20451 (*Fr. sanfranciscensis*), and *Limosilactobacillus reuteri* DSM 20016 (*Lm. reuteri*), were obtained from the DSMZ culture collection (Leibniz Institute DSMZ-German Collection of Microorganisms and Cell Cultures, Germany). All strains were routinely cultivated in appropriate media following the manufacturer's instructions and incubated at 30 °C for 18–24 h, except for *Lm. reuteri*, which was incubated at 37 °C under the same conditions. The selected strains were chosen as type strains representative of phylogenetically and ecologically diverse LAB commonly associated with food fermentation ecosystems. Besides, the use of type strains ensured taxonomic reliability and reproducibility of the comparative analyses.

The cadmium stock solution was prepared by dissolving cadmium chloride (Sigma-Aldrich, St. Louis, MO, USA) in sterile distilled water to obtain a final concentration of 1 g L^−1^. The solution was filter-sterilized through a 0.22 μm membrane filter and stored at 4 °C. Working concentrations were prepared by appropriate dilution of the stock solution in sterile culture medium before each experiment.

The minimum inhibitory concentration (MIC) of cadmium was determined by broth microdilution assay, in accordance with standard guidelines provided by the Clinical and Laboratory Standards Institute (CLSI, 2022). Overnight cultures of LAB were harvested by centrifugation (10,000 × g, 10 min, 4 °C), washed twice with sterile 0.1 M PBS (pH 6.5), and resuspended in fresh medium. The bacterial suspensions were diluted to match a turbidity equivalent to the 0.5 McFarland standard (~1 x 10^6^ CFU/mL). The bacterial inoculum (100 μL) was added to an equal volume of Cd-containing broth in each well with a final volume of 200 μL, to achieve a final inoculum concentration of ~5 x 10^5^ CFU/mL. For each strain, tested cadmium concentrations ranged from 5 ppm to 200 ppm. All MIC determinations were performed in three independent biological replicates, and the values reported in Supplementary Table S1 represent the mean MIC obtained from these experiments. A positive control was included, consisting of bacterial cultures grown in MRS broth without cadmium. The 96-well microplates were incubated at the appropriate temperature, and the optical density (OD) was read at 600 nm before and after incubation. Bacterial growth in cadmium-containing wells was compared with that of the positive control, and the MIC was defined as the lowest cadmium concentration resulting in ≥90% growth inhibition relative to the positive growth control, according to the CLSI guidelines (CLSI, 2022).

### 2.2 Cadmium-induced changes in growth capacity and acidification activity

The physiological response of selected strains was evaluated after exposure to sub-inhibitory cadmium concentrations to assess their adaptation under environmental stress conditions. *W. confusa, Lt. curvatus, Lm. reuteri*, and *Fr. rossiae* were grown in appropriate media for 18 h. Then ~10^7^ cells from each culture were transferred to fresh medium containing cadmium concentrations corresponding to $\frac{1}{8}$, $\frac{1}{4}$, and $\frac{1}{2}$ × MIC and, together with untreated controls, were incubated for 16 h. Further downstream analyses were performed using the same experimental setup.

The pH of the culture medium was measured at the beginning of the experiment (T_0_) and after 16 h of cadmium exposure for each strain using a pH meter (HANNA Instruments, USA). Changes in pH were calculated relative to initial values (T_0_) and used as a proxy for acidification capacity.

For the determination of bacterial growth and survival after cadmium exposure, viable bacteria were quantified by colony-forming unit (CFU) enumeration. After incubation, bacterial cultures were ten-fold serially diluted in sterile physiological saline solution (NaCl 0.9%, *w/v*). Appropriate dilutions were then plated onto the corresponding agar medium and incubated under strain-specific culture conditions. Following incubation, colonies were counted, and bacterial cell density was expressed as log CFU mL^−1^. CFU values for exposed cultures were compared with those of the untreated control to evaluate bacterial growth and survival after exposure.

### 2.3 Microscopic assessment of bacterial cell viability and intracellular lipid remodeling under cadmium stress by LIVE/DEAD and NILE RED

To further characterize the physiological responses of LAB to cadmium exposure, bacterial cultures from the experiments described in Section 2.2 were analyzed by fluorescence microscopy to assess cell viability and alterations in the cellular lipid environment.

The viability of the bacterial cells was determined by using the LIVE/DEAD BacLight Bacterial Viability Kit (L7012, Molecular Probes, USA). Bacterial cells were recovered by centrifugation (10,000 × g, for 10 min), washed twice with phosphate-buffered saline (PBS) 0.05 M, pH 6.5, and finally resuspended in the same buffer to reach an OD value of 0.1 at 600 nm. Following the manufacturer's instructions, cells were stained with SYTO 9 and propidium iodide at a 1:1 ratio and then incubated at 37 °C in the dark for 30 min. Stained bacterial cells were washed three times with PBS and placed in a thin layer of PBS (approximately 2 mm) in 8-well chamber slides.

To assess potential changes in the lipid environment of cells, Nile Red staining was performed on selected lactic acid bacteria strains. Bacterial cell density was adjusted to 0.1 OD_600_ to ensure comparability between treatments. Cells were collected by centrifugation, washed twice with the appropriate culture medium, and stained with Nile Red (Invitrogen, USA) at a final concentration of 1 μg/ml for 15 min in the dark at room temperature. After staining, cells were washed once with culture medium and resuspended in the same medium before imaging to remove excess unbound dye and minimize background fluorescence.

Fluorescence microscopy was performed using a ZEISS LSM880 laser-scanning confocal microscope equipped with an Airyscan detector (Carl Zeiss, Germany). Samples stained with LIVE/DEAD were examined using a 20 × objective, whereas Nile Red-stained samples were examined using a 63 × oil-immersion objective. Autofocus settings, laser power, detector gain, pinhole size, scan speed, and all other acquisition parameters were established before image acquisition and maintained constant throughout the experiment. For each well, five independent positions were automatically selected and recorded. At each position, a z-stack consisting of five optical sections was acquired under identical imaging conditions without manual adjustment between wells or acquisition settings.

For LIVE/DEAD analysis, SYTO 9 and propidium iodide (PI) fluorescence were used to distinguish viable and membrane-compromised bacterial cells. Fluorescence signals were detected within the ranges of 490–560 nm for SYTO 9 and 560–700 nm for PI. For Nile Red analysis, fluorescence signals were collected simultaneously in two spectral channels: 450–605 nm (green channel) and 605–700 nm (red channel).

Image analysis was performed using ZEN 3.11 software (Carl Zeiss, Germany). LIVE/DEAD images were analyzed to determine the proportion of viable and membrane-compromised cells. and quantified as the percentage of dead cells relative to the total bacterial population. Cells were classified as viable (green), non-viable (red), and membrane-compromised (orange/yellow). For Nile Red analysis, bacterial cells were segmented, and fluorescence intensities were measured in both the green and red channels. Mean fluorescence intensity and integrated fluorescence intensity were determined for each channel. To assess cadmium-induced changes in the cellular lipid environment, the green-to-red fluorescence intensity ratio (G/R) was calculated for each image. The fluorescence measurements from the five fields were averaged to obtain a single mean value for each biological replicate, and only these mean values were used for statistical analysis in GraphPad Prism. Quantitative measurements obtained from the five independent positions were averaged for each sample. Identical segmentation and analysis parameters were applied to all experimental groups to ensure comparability among treatments.

### 2.4 Gene loci inspection and retrieval from biological databases

To investigate the genetic basis of cadmium resistance in LAB, genes involved in heavy-metal transport, detoxification, and regulatory pathways were identified through bioinformatics analyses. Gene retrieval was based on the National Center for Biotechnology Information (NCBI) Reference Sequence (RefSeq) database (O'Leary et al., 2016). Candidate loci were identified using the Entrez search system (Kans, 2025) by combining keywords associated with cadmium transport and resistance mechanisms with taxonomic identifiers corresponding to the genus *Lactobacillus* and members of the family Lactobacillaceae. The search strategy employed the following query: (“cadmium resistance” or “cadmium transport” or “cadmium-transporting ATPase” or “c*adA*” or “*cadC*” or “*czc*” OR “*zntA*”) and (“*Lactobacillus*”[Organism] or “*Lactobacillaceae*”[Organism]).

The retrieved gene sequences and their corresponding protein products were manually inspected to verify functional annotation, genomic context, and association with known heavy-metal resistance systems. Particular attention was given to genes encoding P-type ATPases involved in cadmium efflux (CadA/ZntA-like proteins), cation transporters, metal-responsive transcriptional regulators (CadC-family proteins), and components of the Czc metal resistance system. Gene annotations were cross-validated against available RefSeq records and corresponding protein entries.

To confirm functional assignments, predicted protein sequences were analyzed using the NCBI conserved domain database (CDD) (Lu et al., 2020). Conserved domains, catalytic motifs, ATP-binding regions, transmembrane segments, and metal-binding signatures characteristic of heavy-metal-transporting ATPases and associated regulatory proteins were evaluated. Proteins were considered putative cadmium-resistance determinants when they contained conserved domains previously associated with cadmium transport, metal ion homeostasis, or heavy-metal detoxification.

The retrieved information was subsequently used to support target gene selection and the interpretation of phenotypic responses observed during cadmium exposure experiments.

### 2.5 Primer design and target gene validation

Entire gene sequences were used to design primer pairs. Genomic duplications were checked to avoid multiple annealing and to lower the RT-qPCR yields. In particular, primer BLAST software was used for primer design, following the guidelines reported by Ye et al. (2012). Primer specificity, self-complementarity, GC-pair content, melting temperature, and primer-dimer formation potential were assessed *in silico* to ensure optimal amplification efficiency (Supplementary Table S2). Genomic DNA (gDNA) was extracted from the overnight cultures of selected strains using the Wizard Genomic DNA Purification Kit (Promega, Madison, WI, USA) following the manufacturer's instructions. The gDNA was quantified using a NanoDrop 1000 spectrophotometer, and primer pairs were validated in gDNA after PCR with KAPA HiFi HotStart ReadyMix (KAPA Biosystems, Wilmington, MA, USA). PCR products were evaluated using Agilent 2200 TapeStation electrophoresis with D1000 ScreenTape (Agilent Technologies, Santa Clara, CA, USA) to verify specificity and expected amplicon size (~150–200 bp), which is optimal for downstream RT-qPCR and ddPCR applications.

### 2.6 RNA isolation and gene expression analysis through RT-qPCR and ddPCR

Extraction of RNA was carried out on cadmium-exposed and positive control bacterial cells of selected strains after incubation, as described in section 2.2. Cells corresponding to 3.0 OD_600_ were collected by centrifugation. Total RNA was then isolated using an Aurum Total RNA Mini Kit (Bio-Rad, USA) following the manufacturer's instructions. This involves on-column treatment with DNase I to eliminate any traces of gDNA. The quality of RNA in terms of purity, integrity and concentration was measured using an RNA ScreenTape (Agilent Technologies, Santa Clara, CA, USA) on an Agilent TapeStation. Only RNAs with intact ribosomal RNA profiles, devoid of any signs of degradation, were used. Synthesis of complementary DNA (cDNA) was carried out using total RNA (500 ng^−1^ μg) with the iScript gDNA Clear cDNA Synthesis Kit (Bio-Rad, USA), which includes another step of gDNA removal.

For gene expression analysis, quantitative PCR was carried out using a Bio-Rad CFX real-time PCR detection system. SsoAdvanced SYBR Green Supermix (Bio-Rad, USA) was used for the reactions in a final volume of 20 μL with 1x master mix, 0.5 μM of each primer, and 1ng/μL template cDNA. The housekeeping gene *gyr* was used as the internal reference for normalization because of its constitutive expression and its frequent use in LAB gene-expression studies (Eleaume and Jabbouri, 2004). All reactions were carried out in triplicate with no template controls. Relative gene expression was quantified using the comparative Ct value (2^−Δ*ΔCt*^) method) (Livak and Schmittgen, 2001). The gene transcript levels were normalized using the housekeeping *gyr* gene and were expressed in terms of fold change. Data acquisition and analysis were performed using CFX Maestro software. The study was conducted according to the guidelines provided in the MIQE (Bustin et al., 2009). Because preliminary RT-qPCR analyses of *Fr. rossiae* showed low transcript detectability and insufficient amplification efficiency, digital droplet PCR (ddPCR) was used for this strain to enable more sensitive quantification of low-abundance transcripts. ddPCR was performed using the Bio-Rad QX200 Droplet Digital PCR System. The reaction mixture consisted of QX200™ ddPCR™ EvaGreen Supermix (Bio-Rad, USA), 0,5 μM of each forward and reverse primer, and cDNA in each reaction containing 20 μL. The reaction mixture and droplet-generating oil were loaded into the cartridge and partitioned into ~20,000-nanoliter-sized droplets with the QX200™ Droplet Generator (Bio-Rad, USA). The partitioning of the reaction mixture into small droplets allows the digital resolution of the target molecules.

Following droplet generation, the emulsified PCR reactions were placed in a 96-well plate, and it was sealed with a foil heat seal (Bio-Rad, USA) and the PX1™ PCR Plate Sealer (Bio-Rad, USA). The thermal cycling was performed according to the kit instructions. Following amplification, the droplets were analyzed individually using a QX200 droplet reader (Bio-Rad, USA), which measures the fluorescence intensity in each droplet. Analysis of the results was performed using QX Manager Software Standard Edition, in which the droplets are defined as positive or negative, based on fluorescence amplitude thresholds set according to no-template controls and baseline signal distribution. To ensure accurate distinction between positive and negative droplets, manual adjustment of the threshold was performed when necessary.

Absolute quantification of target transcript copies was determined from the percentage of positive droplets, and this was carried out by utilizing Poisson statistics, where the probability of having more than one template in a single droplet was considered (Hindson et al., 2013). The results were expressed as transcript copies/μL of the PCR reaction and subsequently calculated as fold changes relative to the untreated control samples. Each sample was analyzed in three biological replicates, and no-template controls were included in all runs to assess any potential contamination and to set the fluorescence threshold. The reaction was considered valid if it produced >10,000 accepted droplets.

### 2.7 Statistical analysis

All experiments were performed using three independent biological replicates. Data are presented as mean ± standard deviation (SD). Statistical analyses were performed using GraphPad Prism (version 10; GraphPad Software, San Diego, CA, USA). All statistical analyses were performed by a two-way ANOVA using strain and cadmium concentrations as fixed factors, followed by Šídák's multiple-comparisons test. *Post hoc* comparisons were performed between each cadmium-treated group and the corresponding untreated control within each strain. For RT-qPCR analyses, relative gene expression levels were calculated using the 2^−Δ*ΔCt*^ method and statistical comparisons were performed using ΔΔCt values. For ddPCR analyses, fold-change values were compared among treatments using the same statistical approach. Differences were considered statistically significant at *p* < 0.05 (^*^p < 0.05; ^**^p < 0.01; ^***^p < 0.001).

## 3 Results

### 3.1 Cadmium dose–response and strain selection based on minimum inhibitory concentration

Eleven LAB strains were tested for their susceptibility to cadmium concentrations ranging from 0 to 200 ppm in a microplate assay. OD_600_ was measured at T0 and after 16 h of incubation to monitor bacterial growth. Growth inhibition was determined by comparing the OD increase of cadmium-exposed cultures with that of the respective untreated controls. The MIC was defined as the lowest cadmium concentration resulting in ≥90% growth inhibition relative to the control. All tested LAB strains exhibited MIC values ranging from 40 to 160 ppm, with variable dose–response patterns across the taxa panel (Supplementary Table S1).

*Lt. sakei, W. cibaria, L. citreum, Lc. lactis, P. pentosaceus*, and *Lp. plantarum* exhibited non-linear responses to increasing cadmium concentrations, characterized by abrupt increases in growth inhibition between consecutive concentrations. For instance, *W. cibaria* showed a marked increase from 23% inhibition at 40 ppm, to 84% at 80 ppm, while *P. pentosaceus* increased from 42% inhibition at 40 ppm to 97% at 80 ppm. Similarly, *Lp. plantarum* displayed a sharp transition from 20% inhibition at 10 ppm to 71% at 20 ppm. These sharp transitions limited the identification of physiologically distinct sub-inhibitory conditions suitable for subsequent analyses. In contrast, *Fr. sanfranciscensis* displayed high sensitivity even at low cadmium concentrations, reaching 48%, 86%, and 89% inhibition at 5, 10, and 20 ppm, respectively, and exceeding the MIC threshold (90% inhibition) at 40 ppm, thus limiting the definition of consecutive sub-inhibitory exposure levels.

On the other hand, *Fr. rossiae* and *Lt. curvatus* exhibited MIC values of 80 ppm and showed a gradual increase in growth inhibition with increasing cadmium concentrations. At sub-inhibitory levels corresponding to $\frac{1}{8}$, $\frac{1}{4}$, and $\frac{1}{2}$ × MIC (10, 20, and 40 ppm), inhibition increased from 10% to 36% and 69% in *Fr. rossiae*, and from 31% to 57% and 86% in *Lt. curvatus*. A similar dose-dependent pattern was observed in *W. confusa* and *Lm. reuteri*, both characterized by a MIC of 160 ppm. At $\frac{1}{8}$, $\frac{1}{4}$, and $\frac{1}{2}$ × MIC (20, 40, and 80 ppm), growth inhibition reached 9%, 32%, and 67% in *W. confusa*, and 37%, 64%, and 78% in *Lm. reuteri*.

Therefore, *Fr. rossiae* (DSM 15814*), Lt. curvatus* (DSM 20019*), W. confusa* (DSM 20196), and *Lm. reuteri* (DSM 20016) were selected for subsequent physiological, microscopic, and transcriptional analyses (Figure 1).

**Figure 1:**
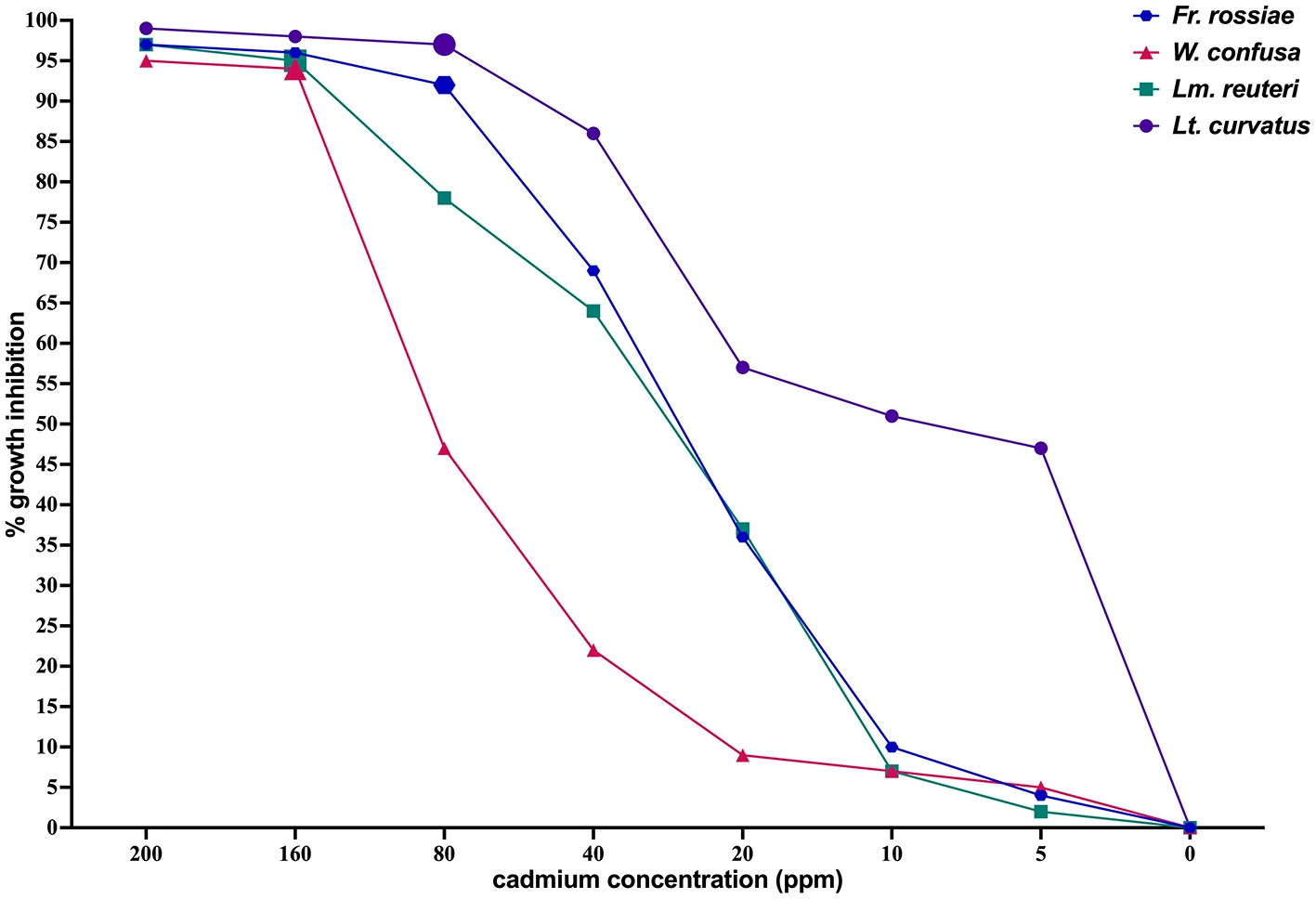
Cadmium dose–response profiles of selected lactic acid bacteria. Percentage growth inhibition of *Fr. rossiae* (blue), *W. confusa* (red), *Lm. reuteri* (green), and *Lt. curvatus* (purple) following exposure to increasing concentrations of cadmium (0–200 ppm). Growth inhibition was calculated relative to the untreated control. Each symbol represents the corresponding LAB strain, as indicated in the legend.

### 3.2 Physiological responses to cadmium treatment: cell viability, cultivability, pH dynamics, and cellular lipid environment

The physiological impact of exposure to sub-lethal cadmium concentrations was evaluated on the four selected strains by assessing acidification rates and cell viability, the latter determined through both plate counts and live/dead.

The pH value measurements showed significant strain-dependent changes (Figure 2A). After 16-h incubation, *W. confusa* maintained a remarkably stable pH (4.35) under all conditions, suggesting that metabolic activity was unaffected by cadmium administration. All the other strains showed higher pH values at increasing cadmium concentrations, suggesting a stronger reduction in metabolic activity. This effect was most pronounced in *Lm. reuteri*, where the final pH increased from 4.2 in the untreated control to 4.75 at $\frac{1}{2}$ × MIC. Smaller increases were observed in *Fr. rossiae* (4.4 to 4.64) and *Lt. curvatus* (4.3 to 4.64) under the same conditions.

**Figure 2:**
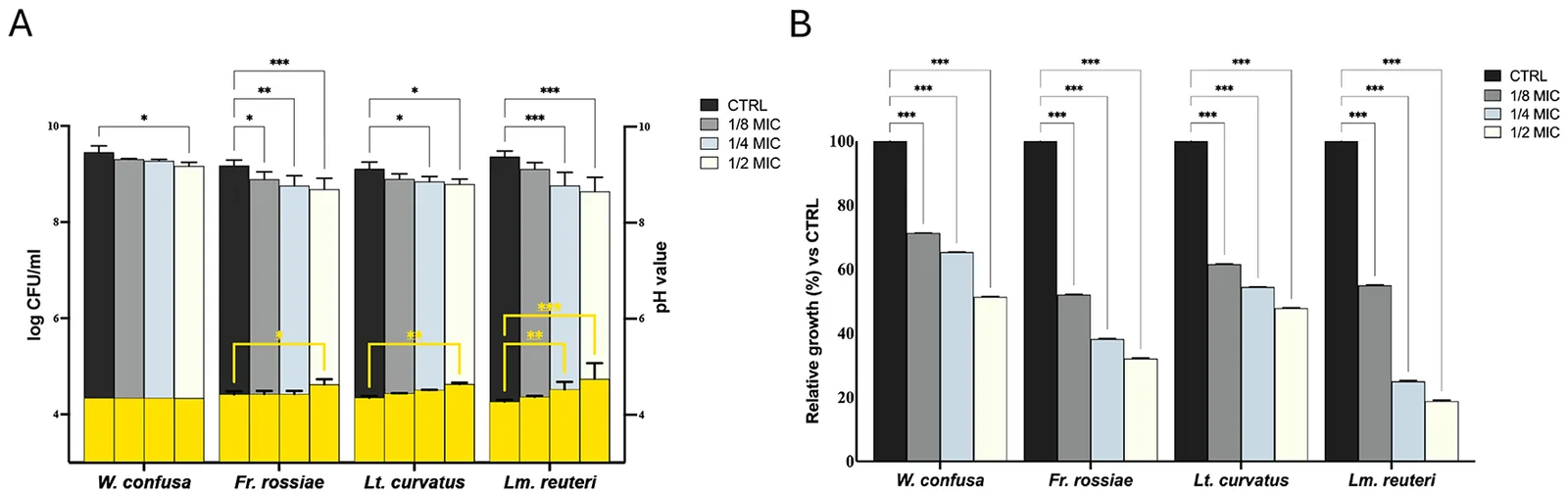
Dose-dependent suppression of bacterial growth under cadmium stress. **(A)** Bacterial density in terms of log CFU/ml and modulation of culture medium pH after 16 h of cadmium exposure at 1/8, 1/4, and 1/2 MIC. **(B)** Normalized relative growth (%) of bacterial strains based on computed percentage of raw density values. Yellow bars indicate final pH values (right y-axis), whereas blue bars represent cultivability (left y-axis). The data are given as mean ± SD for three experiments. Significance: *P* < 0.05 (*), *P* < 0.01 (**), *P* < 0.001 (***).

After cadmium exposure, LABs' cell density consistently declined in a concentration-dependent manner (Figure 2A). *W. confusa* maintained the highest CFU values under all tested conditions, only decreasing from 9.46 log CFU mL^−1^ in the control to 9.17 log CFU mL^−1^ at $\frac{1}{2}$ × MIC, reinforcing its classification as a tolerant phenotype. In contrast, *Lm. reuteri* exhibited the most pronounced reduction, declining from 9.36 to 8.64 log CFU mL^−1^ across the same conditions, suggesting a decreased ability to maintain cellular function under stress. *Fr.rossiae* and *Lt.curvatus* showed intermediate responses, with CFU values decreasing in control and at $\frac{1}{2}$ × MIC from 9.18 to 8.68 log CFU mL^−1^ and from 9.11 to 8.79 log CFU mL^−1^, respectively, indicating intermediate loss in culturability (Figure 2A). Although the differences in cell density did not reach a full log cycle, strain-specific effects were more evident when growth was expressed relative to the untreated control, highlighting the percentage decrease in viable cells. As reported in Figure 2B, significant inhibition of growth was observed even at the lowest sub-inhibitory cadmium concentrations ($\frac{1}{8}$ × MIC), and it was more pronounced at higher cadmium concentrations ($\frac{1}{4}$ and $\frac{1}{2}$ × MIC). *W. confusa* retained the highest growth capacity, decreasing to 71.3%, 65.4%, and 51.4% at $\frac{1}{8}$, $\frac{1}{4}$, and $\frac{1}{2}$ × MIC, respectively. In contrast, *Lm.reuteri* exhibited the strongest reduction, with relative growth declining to 55.0%, 25.0%, and 18.8% across the same conditions. *Fr.rossiae* and *Lt.curvatus* showed intermediate responses, decreasing up to 32.1% and 47.9% at $\frac{1}{2}$ × MIC, respectively.

Live/Dead staining and confocal laser scanning microscopy techniques were thus adopted to highlight cell viability changes after 16 h of cadmium exposure (Figure 3A). *Fr. rossiae* showed dead cells increasing from 14.3% in the control to 23.2%, 53.8%, and 77.6% at $\frac{1}{8}$, $\frac{1}{4}$, and $\frac{1}{2}$ × MIC, respectively. In contrast, *W. confusa* showed a more gradual increase, rising from 11.9% to 26.4%, 39.1%, and 47.4% across the same conditions, indicating a greater ability to maintain membrane integrity (Figure 3B). *Lm. reuteri* and *Lt. curvatus* displayed comparatively lower levels of cell death. *Lm. reuteri* increased from 5.3% to 11.1%, 12.9%, and 17.4%, whereas *Lt. curvatus* showed the lowest overall impact, with values rising from 1.7% in the control to 5.4%, 5.8%, and 19.4% at increasing cadmium concentrations (Figure 3B).

**Figure 3:**
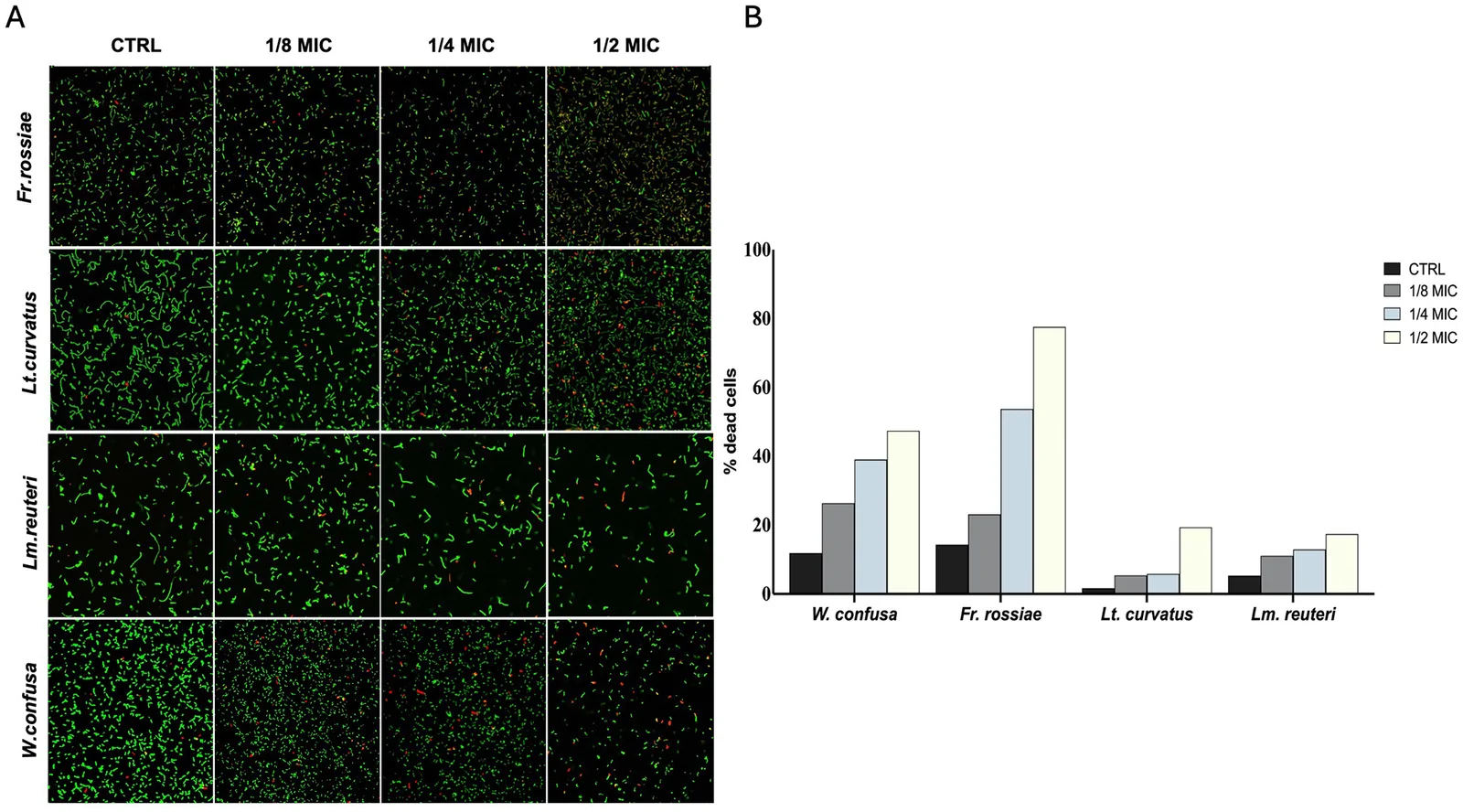
Cadmium-induced loss of bacterial viability, as determined by Live/Dead staining combined with confocal laser scanning microscopy (CLSM). **(A)** Representative CLSM images of Live/Dead-stained cells of *selected four LABs* after 16 h of cadmium exposure at 1/8, 1/4, and 1/2 MIC. Viable cells are stained green (SYTO 9), whereas cells with compromised membrane integrity are stained red (propidium iodide). **(B)** Quantification of cell death expressed as a percentage of non-viable cells based on fluorescence analysis.

Nile Red fluorescence analysis revealed marked changes in the cellular lipid environment of all investigated LAB strains following cadmium exposure (Figure 4). The green-to-red fluorescence ratio (G/R), expressed as fold change relative to the untreated control, increased in all strains, although the magnitude of the response varied depending on both strain and cadmium concentration. In *Fr. rossiae*, the G/R ratio increased by 1.94-, 2.64-, and 2.67-fold following exposure to 10, 20, and 40 ppm cadmium. A similar trend was observed for *Lt. curvatus*, which exhibited increases of 2.84-, 2.92-, and 2.97-fold at 10, 20, and 40 ppm, respectively (Figure 4B). In contrast, *W. confusa* displayed a more gradual response to cadmium exposure. The G/R ratio increased by 1.33-fold at 20 ppm, reaching 1.91- and 2.10-fold at 40 and 80 ppm, respectively. Among all strains analyzed, *Lm. reuteri* exhibited the strongest response. While moderate increases were observed at 20 ppm (1.60-fold) and 40 ppm (1.72-fold), exposure to 80 ppm resulted in a pronounced increase in the G/R ratio, reaching a 5.61-fold change relative to the control (Figure 4B).

**Figure 4:**
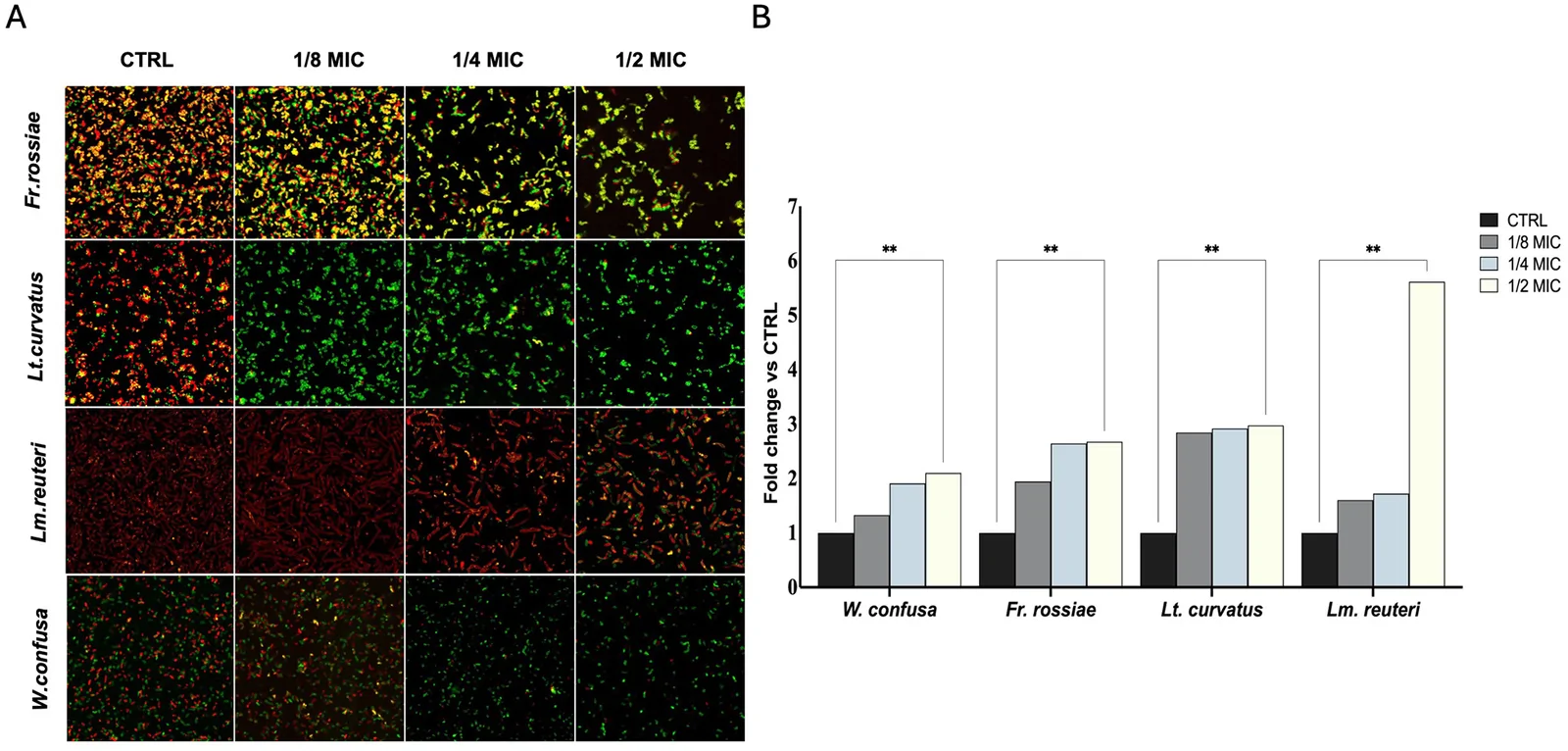
Cadmium-induced changes in membrane lipid organization of selected LAB determined by CLSM. **(A)** Representative confocal microscopy images of Nile Red-stained cells following exposure to cadmium at 1/8, 1/4 and 1/2 × MIC. Pseudocolours represent the relative distribution of Nile Red fluorescence and provide a qualitative visualization of cadmium-induced changes in the membrane lipid environment. The corresponding green-to-red (G/R) fluorescence ratios shown in **(B)** were obtained by quantifying the fluorescence intensities of the green and red channels using the confocal microscope image analysis software. **(B)** Nile Red G/R fluorescence ratio expressed as fold change relative to the untreated control (CTRL). Significance: *P* < 0.05 (*), *P* < 0.01 (**), *P* < 0.001 (***).

### 3.3 From the *in silico* analysis of genomic loci to obtain gene amplicons

Candidate genes involved in cadmium resistance, detoxification, and metal homeostasis were selected to cover fundamental functional elements of the bacterial adaptation process to metal stress, in line with previous literature (Table 1) (Nies, 2003; Chandrangsu et al., 2017; Li et al., 2021). The inspection of genomic annotation data based on RefSeq NCBI genomes relative to our DSMZ strains revealed the presence of individual genes rather than a complete operon structure. These include the *cadA* gene, which encodes an ATP-dependent efflux system (Solovieva and Entian, 2002; Helbig et al., 2008), and the *czcA* gene, which encodes an RND-type transporter, both involved in the active efflux of toxic metal ions and reduction of intracellular cadmium burden (Nies, 2003; Liu et al., 2021). *W. confusa* was found to possess mntH as the major gene associated with metal transport-related functions. Notably, *F. rossiae* possessed a range of gene traits, including *czcA, cadD*, and *mntH*, which are associated with resistance, thus pinpointing a more complex stress adaptation strategy. Supplementary Figure S1 shows the primer performance electrophoretic validation on the TapeStation system, after PCR reaction using gDNA obtained from each strain as a template.

**Table 1:** Functional overview of genes involved in bacterial cadmium resistance pathways.

Gene	Operon	Location	Biological function	Main metals	Mechanism of action	Protein type/family	Functional redundancy	Regulation
*cadA*	*cad* operon (*cadA*–*cadC*/*cadD*)	Chromosome/ plasmid	Detoxification of toxic metals	Cd^2^^+^, Zn^2^^+^	ATP-driven efflux pump exporting toxic metals	P-type ATPase transporter	Overlaps with zntA in metal detoxification	Metal-responsive regulator (CadC)
*cadD*	*cad* operon	Chromosome/ plasmid	Cadmium resistance protein supporting efflux system	Cd^2^^+^	Membrane protein contributing to cadmium resistance	Cadmium resistance protein	Partial overlap with cadA	cad operon regulators
*czcA*	*czcCBA* operon	Chromosome	Heavy-metal detoxification	Co^2^^+^, Zn^2^^+^, Cd^2^^+^	RND-type efflux system exporting metals across membranes	RND efflux transporter	Alternative efflux system	Metal-responsive regulators
*mntH*	*mntH* transport system	Chromosome	Manganese uptake and metal homeostasis	Mn^2^^+^, Cd^2^^+^	Proton-dependent metal ion transport into the cell	NRAMP family transporter	Redundant with other Mn transport systems	MntR/Fur regulators

The operon organization reported in this table represents the canonical architecture described in the literature for bacterial cadmium-resistance systems and it is provided as a functional reference. Genome annotation of the LAB strains analyzed in this study identified individual resistance-associated genes rather than complete operon structures; therefore, the operon arrangements shown do not represent the genomic organization of the strains investigated.

### 3.4 Transcriptional profiling of selected cadmium resistance traits under cadmium exposure

The integrity of total RNA was confirmed by RIN values before gene expression analysis by electrophoretic TapeStation, with all samples exhibiting intact profiles and no detectable degradation (Supplementary Figure S2). The stability of the reference gene *gyr* was evaluated across all cadmium treatments for the RT-qPCR experiments (*W. confusa, Lt. curvatus, and Lm. reuteri*). The Ct values showed minimal variation among experimental conditions (Supplementary Table S3), confirming that *gyr* remained stably expressed and was suitable for normalization under the conditions investigated. For *Fr. rossiae*, transcript quantification was performed by ddPCR, which provides absolute transcript quantification and therefore did not require normalization to the *gyr* reference gene. In *W. confusa, mntH* gene showed a progressive and statistically significant upregulation in response to rising cadmium concentrations, with the highest fold-change observed at the $\frac{1}{2}$ × MIC treatment (1.75-fold relative to the CTRL; Figure 5A). In *Lt. curvatus*, both *mntH* and *cadA* exhibited a non-monotonic transcriptional response across the sub-inhibitory concentrations tested. Specifically, *cadA* reached its maximum up-regulation at the lowest tested cadmium concentration ($\frac{1}{8}$ × MIC, showing a 1.40-fold increase relative to the CTRL), while *mntH* peaked at $\frac{1}{4}$ × MIC, with a 1.31-fold increase. Notably, at the highest cadmium concentration tested ($\frac{1}{2}$ × MIC), the expression of both genes declined to levels not statistically significant compared to the control (1.03 and 1.04, respectively; Figure 5B). On the other hand, in *Lm. reuteri mntH* exhibited strong and highly significant up-regulation at both $\frac{1}{8}$ and $\frac{1}{4}$ × MIC, with the peak induction occurring at the lowest concentration ($\frac{1}{8}$ MIC −4.2 fold-change). In contrast, *cadD* showed significant induction only at $\frac{1}{4}$ × MIC (2.5-fold change). For both genes, transcriptional activity at $\frac{1}{2}$ × MIC was comparable to that observed in the control (Figure 5C). In the case of *F. rossiae*, quantification of the genes of interest was not possible by RT-qPCR, possibly due to low transcript levels and inefficient amplification, which resulted in a lack of detection. Therefore, ddPCR was employed for the evaluation of gene expression in *Fr. rossiae*, as it enables sensitive and absolute quantification of low-abundance transcripts. Although results are expressed as fold change relative to controls for both RT-qPCR and ddPCR, the two methods rely on fundamentally distinct quantification principles (Ct-based relative quantification vs. partition-based absolute quantification) and exhibit different dynamic ranges. Accordingly, fold-change magnitudes are not directly comparable between *Fr. rossiae* (ddPCR) and the other strains (RT-qPCR), and cross-strain comparisons of expression amplitude should be interpreted with caution.

**Figure 5:**
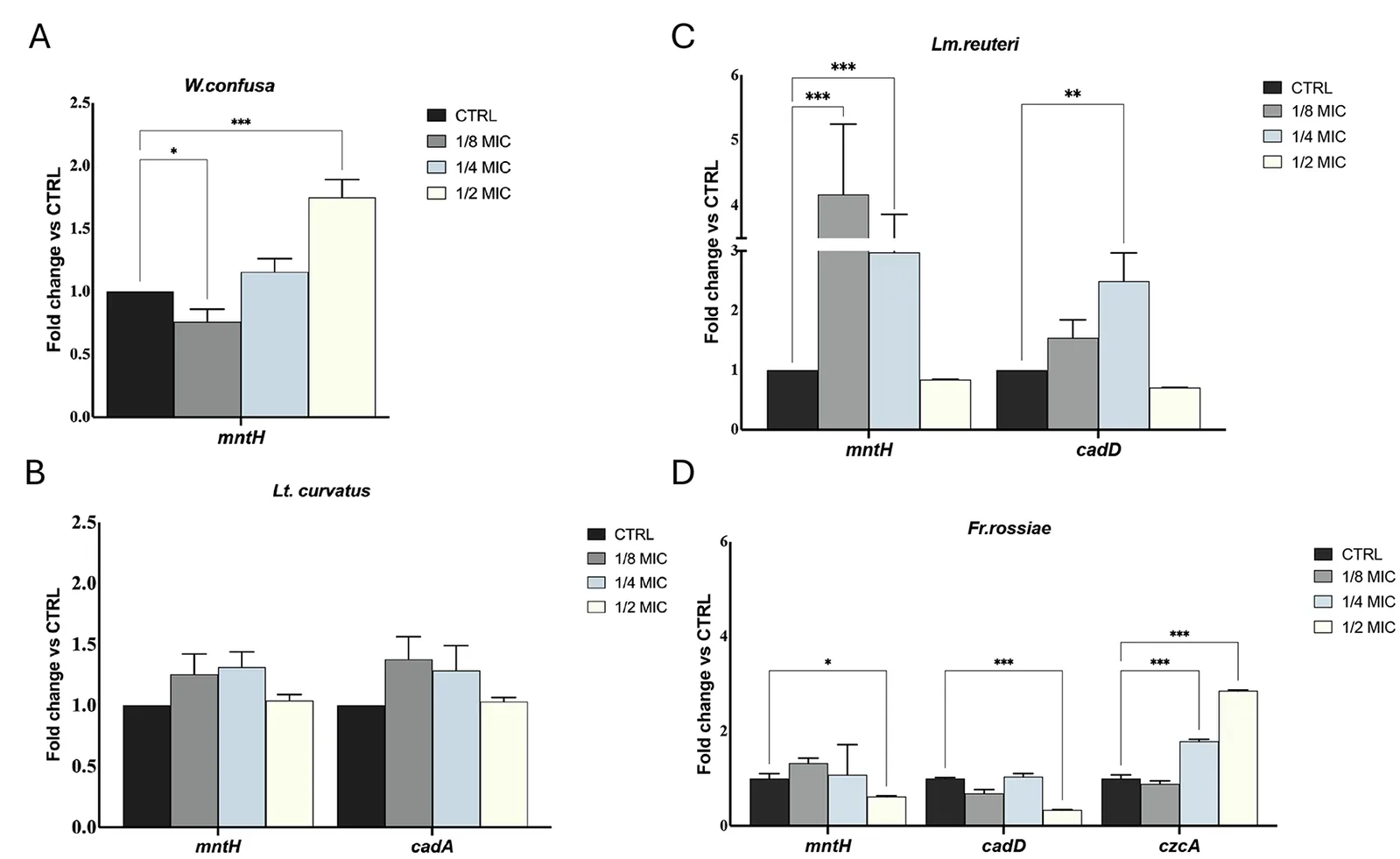
Transcriptional response of cadmium resistance determinants under cadmium exposure. Relative expression of selected cadmium resistance genes in *W. confusa* **(A)**, *Lt. curvatus* **(B)**, *Lm. reuteri* **(C)**, and *Fr. rossiae* **(D)** after 16 h of cadmium exposure at concentrations of 1/8, 1/4, and 1/2 MIC. The expression levels of the cadmium resistance genes were normalized relatively to the control. The expression of cadmium resistance genes was analyzed by RT-qPCR, whereas for *Fr. rossiae*, which has low levels of transcripts, ddPCR was employed. The error bars indicate the SD of three independent experiments. The statistical significance of the data relative to the control is denoted as *P* < 0.05 (*), *P* < 0.01 (**), and *P* < 0.001 (***).

In *Fr. rossiae*, cadmium exposure induced gene-specific and concentration-dependent transcriptional responses. *mntH* and *cadD* were significantly downregulated at $\frac{1}{2}$×MIC, while czcA was significantly upregulated at $\frac{1}{4}$×MIC and $\frac{1}{2}$×MIC (Figure 5D).

## 4 Discussion

The present study was aimed at investigating the physiological and transcriptional adaptation mechanisms across LAB under cadmium sub-inhibitory stress conditions. First, our methodological approach relied on the selection of eleven LAB type strains from different species, representative of specific fermented food niches. The initial screening of the eleven strains was based on MIC calculations, which revealed considerable variation in cadmium susceptibility, with values ranging from 40 to 160 ppm and relatively highly variable dose-response patterns. Such heterogeneity is consistent with previous studies reporting that bacterial heavy-metal resistance is strongly influenced by species-specific physiological traits, ecological origin, and the repertoire of metal resistance determinants encoded within the genome (Bruins et al., 2000; Nies, 2003). The great majority of the tested strains did not exhibit a progressive response to increasing cadmium concentrations. In fact, *Lt. sakei, W. cibaria*, and *L. citreum, Lp. plantarum, Lc. lactis*, and *P. pentosaceus* showed marked variations in growth inhibition between consecutive concentrations, suggesting a narrow transition between adaptive and inhibitory responses. These discontinuous patterns can complicate the identification of physiologically meaningful sub-inhibitory concentrations suitable for downstream analyses. In contrast, *Fr. rossiae, Lt. curvatus, W. confusa* and *Lm. reuteri* exhibited gradual and linear dose-dependent responses, allowing the reliable establishment of $\frac{1}{8}$, $\frac{1}{4}$, and $\frac{1}{2}$ × MIC conditions. Consequently, only these four strains were selected as representative models for investigating the physiological and molecular mechanisms.

The initial screening of the eleven LAB strains revealed substantial variability in cadmium susceptibility (MIC 40–160 ppm) and also in shaping their dose–response patterns. Taken alone, MIC values did not capture the continuity of bacterial responses across sub-inhibitory concentrations and were not indicative of increased physiological stress as a result of progressively higher exposures to cadmium. Such heterogeneity is consistent with previous studies reporting that bacterial heavy-metal resistance is strongly influenced by species-specific physiological and genomic traits, rather than ecological origin (Bruins et al., 2000; Nies, 2003). Specifically, most strains monitored in this study exhibited discontinuous dose–response profiles, featuring abrupt transitions in growth where small increases in cadmium concentration produced disproportionate reductions in bacterial growth. This prevents the establishment of a controlled physiological stress gradient. This was not the case for *Fr. rossiae, Lt. curvatus, W. confusa*, and *Lm. reuteri* that displayed continuous dose-dependent inhibition at each successive step in growth reduction. Therefore, these four strains were selected based on the robustness and interpretability of their dose–response behavior rather than on their absolute level of cadmium tolerance.

*W. confusa* and *Lm. reuteri* displayed the highest MIC values (160 ppm), whereas *Fr. rossiae* and *Lt. curvatus* showed intermediate resistance levels (80 ppm). However, despite exhibiting similar MIC values, strains responded differently in terms of metabolic activity, membrane integrity, lipid remodeling, and transcriptional regulation.

Following 16 h of cadmium exposure, all four strains exhibited reduced acidification capacity, indicating that fermentative metabolism was highly sensitive to sub-inhibitory cadmium stress under the experimental conditions investigated. Although bacterial growth was only moderately affected at lower sub-inhibitory concentrations, significant increases in final pH values were consistently observed, suggesting that cadmium impaired fermentative metabolism even under conditions where reductions in bacterial growth remained relatively limited. Similar observations have been reported in *Bacillus subtilis* and *Escherichia coli*, where cadmium disrupts glycolysis, energy metabolism, and intracellular redox balance through interactions with thiol-containing enzymes and metal-dependent proteins (Chandrangsu et al., 2017; Li et al., 2021). Collectively, these findings indicate that acidification activity represents a sensitive physiological indicator of cadmium stress. However, because all physiological measurements were performed after a single 16 h exposure, the present study does not establish whether metabolic alterations precede reductions in cultivability or occur concomitantly as part of the overall adaptive response.

The plate count data further demonstrated that increasing cadmium concentrations progressively reduced bacterial cultivability; however, reductions in cultivability were not always accompanied by proportional losses in viability. While *Lm. reuteri* showed the largest reduction in CFU counts, LIVE/DEAD analysis revealed only moderate levels of membrane damage. On the other hand, *Fr. rossiae* exhibited the highest proportion of membrane-compromised cells despite showing a less pronounced reduction in cell density. These findings indicate that growth inhibition and membrane damage represent distinct aspects of cadmium toxicity and suggest that loss of culturability may occur before their membrane are permanently destroyed. However, the persistence of membrane-intact cells with reduced culturability, assessed by plate counts and LIVE/DEAD staining, means that the current data do not allow discrimination between temporarily growth-impaired cells and truly viable but nonculturable VBNC-like cell subpopulations, which would require additional evidence of cellular recovery, metabolic activity, or resuscitation capacity (Oliver, 2010; Ayrapetyan et al., 2015; Highmore et al., 2018).

Nile Red fluorescence analyses provided additional insights into adaptive stress responses occurring at the membrane level. All the investigated strains exhibited increased green-to-red (G/R) fluorescence ratios following cadmium treatment, indicating significant alterations in the bacterial membrane lipid environment. Because Nile Red is a solvatochromic dye, its fluorescence changes according to the polarity of the surrounding lipid environment and it is influenced by its physicochemical characteristics. In bacteria lacking the large neutral lipid droplets characteristic of eukaryotic cells, changes in Nile Red fluorescence can be more appropriately interpreted as reflecting alterations in membrane lipid organization and physicochemical properties, including phospholipid packing, membrane fluidity, lipid order and membrane hydrophobicity (Strahl et al., 2014), which may arise from stress-induced modifications in membrane fatty acid composition and phospholipid organization reported in bacteria exposed to environmental stressors (Zhang and Rock, 2008; Siliakus et al., 2017). Consequently, the increased G/R ratios observed in the present study are consistent with cadmium-associated alterations in the membrane lipid environment and may indicate adaptive membrane-associated responses involved in maintaining cellular homeostasis under metal stress.

Importantly, the magnitude of the changes in the membrane lipid environment differed considerably among strains and closely reflected their physiological responses. Our results showed how *W. confusa* maintained the highest cultivability and relatively low levels of membrane damage, exhibiting only moderate changes in the G/R ratio. In contrast, *Lm. reuteri* displayed a dramatic increase in the G/R ratio, exceeding fivefold at the highest cadmium concentration. This pronounced membrane remodeling coincided with the greatest reduction in growth performance, suggesting that the extent of membrane adaptation reflects the severity of cadmium-induced physiological stress rather than enhanced resistance *per se*. Similarly, *Fr. rossiae* and *Lt. curvatus* exhibited stable two- to threefold increases in the G/R ratio, indicating activation of membrane adaptation mechanisms even under moderate cadmium exposure.

On the other hand, the transcriptional analyses we provided offered the chance to inspect these physiological observations.

Due to the non-comparable assets in the considered strains, the analyzed gene panels were intentionally selected according to the repertoire of cadmium-associated genes identified through the *in silico* genome annotation. Consequently, it is not possible to compare the expression magnitude of identical resistance determinants across LAB species. Instead, our transcriptional analyses were intended to investigate how each strain modulates its own genetically encoded metal-homeostasis and detoxification machinery in response to cadmium stress. Therefore, comparisons among strains should be interpreted qualitatively, reflecting distinct adaptive strategies associated with different genomic backgrounds rather than direct comparisons of equivalent regulatory circuits.

In *W. confusa*, the progressive upregulation of *mntH* across increasing cadmium concentrations indicates a coordinated homeostatic response that likely contributes to maintaining intracellular metal balance during cadmium stress. *MntH* transporters are known to participate in divalent metal transport and protection against metal-induced oxidative stress through the regulation of intracellular manganese availability (Kehres and Maguire, 2003; Chandrangsu et al., 2017; Bosma et al., 2021). Consistent with this role, the sustained induction of *mntH* suggests a contribution to the maintenance of intracellular metal homeostasis during cadmium stress and plausibly supports the high physiological resistance observed in *W. confusa*.

In *Lt. curvatus* and *Lm. Reuteri*, the analyzed resistance-associated genes were induced primarily at low and intermediate cadmium concentrations. The induction of *cadA, cadD*, and *mntH* suggests effective activation of detoxification pathways under moderate cadmium stress. Similar responses have been reported in *Listeria monocytogenes* and *Bacillus subtilis*, where cadmium resistance systems are effectively induced under moderate metal stress but become less efficient at higher toxicant concentrations due to the increasing energetic demands associated with metal efflux, maintenance of intracellular homeostasis, and cellular stress responses (Helbig et al., 2008; Helmann, 2014).

*Fr. rossiae* displayed a particularly divergent transcriptional pattern. While *mntH* and *cadD* expression decreased significantly at the highest cadmium concentration, *czcA* expression increased progressively. These expression patterns suggest an increased contribution of active metal extrusion at higher cadmium concentrations. The combined physiological, membrane-associated, and transcriptional responses observed in *Fr. rossiae* suggest that increasing cadmium concentrations progressively exceed the capacity of homeostatic mechanisms to maintain cellular equilibrium. Based on the integration of the physiological, membrane-associated and transcriptional responses, active metal extrusion is hypothesized to become increasingly important in *Fr. rossiae* as intracellular cadmium stress intensifies. However, the transcriptional changes observed in this study are interpreted as indicators of regulatory adaptation to cadmium exposure rather than as direct evidence of transporter activation or functional resistance mechanisms, because transcript abundance alone does not necessarily reflect protein abundance, enzymatic activity, or metal transport capacity.

Overall, our results indicate that LAB respond to cadmium stress through a combination of physiological changes, membrane adaptation, and regulation of resistance-related genes. Although some strains showed similar cadmium tolerance, their response mechanisms differed considerably, suggesting that multiple adaptation strategies exist. These findings suggest that LAB adaptation to cadmium stress is associated with the regulation of resistance-related genes and changes in cellular physiological plasticity.

Overall, the transcriptional responses were quantified using two complementary molecular approaches. ddPCR was employed for *Fr. rossiae* because transcript abundance was below the detection limits of RT-qPCR, whereas RT-qPCR was used for the remaining strains. Although both methods provide robust measurements of relative gene expression, their distinct analytical principles mean that fold-change magnitudes are not directly equivalent. Accordingly, comparisons between *Fr. rossiae* and the other strains should be interpreted qualitatively, focusing on biologically relevant expression patterns rather than on the absolute magnitude of transcriptional induction.

## 5 Conclusions

This comparative and exploratory study was designed to investigate the physiological and transcriptional responses of lactic acid bacteria strains to cadmium exposure under sub-inhibitory stress conditions. An initial panel of eleven LAB strains, selected as representative food-associated lactobacilli, was screened for cadmium tolerance. Among them, four strains displaying linear and progressive dose–response patterns were selected for further analyses, allowing the investigation of strain-specific adaptation patterns under comparable sub-inhibitory cadmium conditions. The results demonstrated a marked strain-dependent variability in cadmium tolerance, both at physiological and molecular levels. Even sub-inhibitory levels of cadmium significantly affected bacterial metabolism, impairing acidification activity and, to a lesser extent, bacterial growth. The increase in final pH values observed under higher sub-inhibitory conditions suggests that alterations in fermentative activity occur at early stages of cadmium stress, preceding reductions in culturability and viability. Transcriptional analyses further revealed heterogeneous expression patterns among strains. Although genes associated with metal transport, efflux, and homeostasis (e.g., *cadA/cadD* and *mntH)* were generally induced in the presence of cadmium, their activation occurred at different exposure levels depending on the strain, indicating that cadmium adaptation is likely regulated through strain-specific physiological and regulatory dynamics rather than gene presence alone. Importantly, *in silico* genomic analyses highlighted the limited and fragmented annotation of cadmium-related determinants in LAB genomes, with the absence of conserved and complete operon structures across the investigated strains. This limitation restricted the possibility of establishing direct genotype–phenotype correlations and underlines the need for broader genomic and omics-based investigations to better elucidate the molecular mechanisms underlying bacterial adaptation to metal stress. Overall, these findings indicate that LABs responding to cadmium are associated with strain-specific physiological and regulatory traits, highlighting the relevance of sub-inhibitory metal exposure in shaping microbial functionality and stress-response patterns. Further omics-based approaches will elucidate specific pathways and provide deeper insight into the mechanisms underlying stress adaptation to metal-enriched niches.

## Data Availability

The datasets presented in this study can be found in online repositories. The names of the repository/repositories and accession number(s) can be found in the article/Supplementary material.
