# Supplementary material for: Physiological and transcriptional responses of lactic acid bacteria to sub-inhibitory cadmium exposure
Source: Front Microbiol. 2026 Oct 13;17:1914052. doi: 10.3389/fmicb.2026.1914052 (PMC13712412; doi:10.3389/fmicb.2026.1914052)

Figure S1. Validation of primer specificity by electroforetic TapeStation analysis. To check the primer specificity, PCR products of gDNA of *Lm. reuteri, Lt. curvatus, W. confusa*, and *Fr. rossiae* using gene-specific primers were analysed by band sizes using an automated capillary electrophoresis TapeStation D1000.

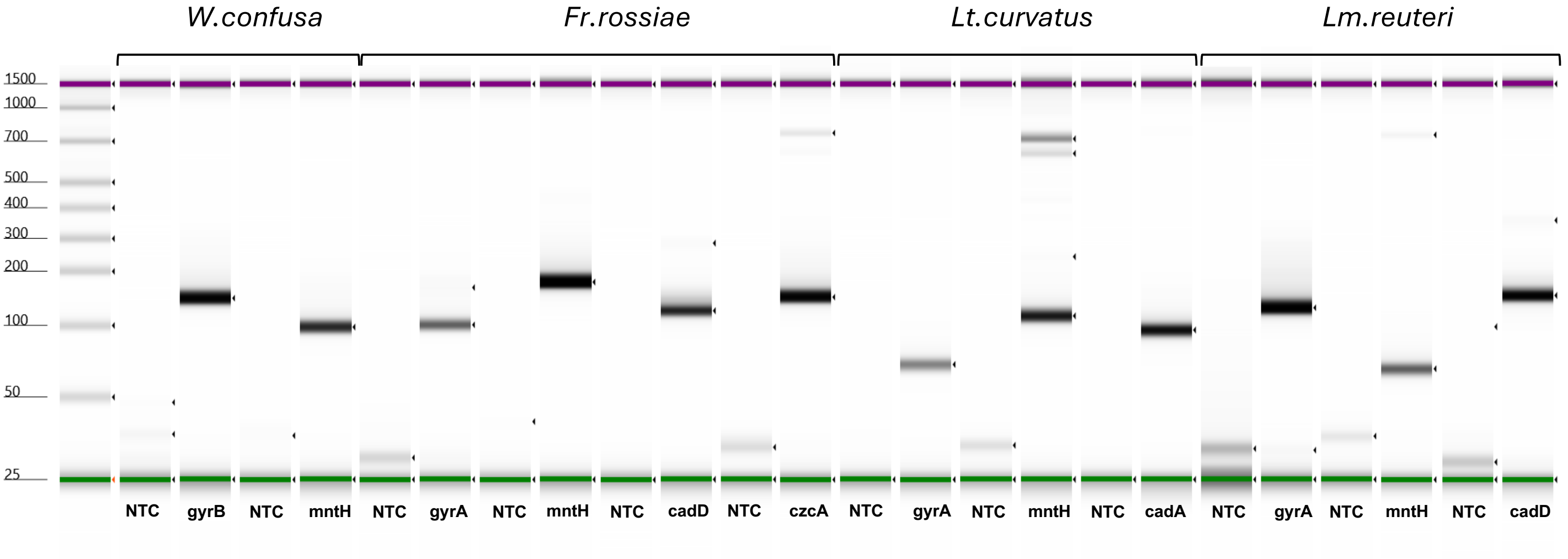

Supplement: Supplementary file 1 [file Image_1.pdf]
