# Supplementary material for: Physiological and transcriptional responses of lactic acid bacteria to sub-inhibitory cadmium exposure
Source: Front Microbiol. 2026 Oct 13;17:1914052. doi: 10.3389/fmicb.2026.1914052 (PMC13712412; doi:10.3389/fmicb.2026.1914052)
Supplement: Supplementary file 3 [file Table_1.docx]

Table S1. Dose-dependent growth inhibition (%) of bacterial taxa across cadmium concentrations (ppm). Growth inhibition rates derive from triplicate measurements. Asterisks (*) indicate the minimum inhibitory concentration (MIC), defined as the lowest cadmium concentration resulting in ≥90% growth inhibition relative to the untreated control. Underlined values indicate the sub-inhibitory cadmium concentrations (1/8, 1/4, and 1/2 × MIC) selected for subsequent physiological and transcriptional analyses.

| **Cadmium concentration** | ***Lt. sakei***  *(DSM 20017)* | ***W. cibaria***  *(DSM 14295)* | | ***L. citreum***  *(DSM 5577)* | ***Lp. plantarum***  *(DSM 20174)* | | ***P. pentosaceus***  *(DSM 20336)* | | ***Lc. lactis***  *(DSM 20481)* | | ***Fr. sanfranciscensis***  *(DSM 20451)* | | | ***Lm. reuteri***  *(DSM 20016)* | | ***W. confusa***  *(DSM 20196)* | | ***Lt. curvatus***  *(DSM 20019)* | | ***Fr. Rossiae***  *(DSM 15814)* | |
| --- | --- | --- | --- | --- | --- | --- | --- | --- | --- | --- | --- | --- | --- | --- | --- | --- | --- | --- | --- | --- | --- |
| **Origin of strains** | “Moto" starter  of sake | kimchi (Korean fermented vegetable) | | honey dew of rye ear | pickled cabbage | | dried American  beer yeast | | unknown | | San Francisco  sourdough | | intestine of adult | | | sugar cane | | milk | | wheat sourdough | |
| ***200 ppm*** | 95% | 94% | 92% | | 92% | 100% | | 97% | | 100% | | 97% | | | 95% | | 99% | | 97% | |  |
| ***160 ppm*** | ***94%** | ***93%** | ***91%** | | 92% | 99% | | ***95%** | | 93% | | ***95%** | | | ***94%** | | 98% | | 96% | |  |
| ***80 ppm*** | 88% | 84% | 81% | | ***91%** | ***97%** | | 86% | | 91% | | 78% | | | 47% | | ***97%** | | ***92%** | |  |
| ***40 ppm*** | 72% | 23% | 42% | | 83% | 42% | | 19% | | ***90%** | | 64% | | | 22% | | 86% | | 69% | |  |
| ***20 ppm*** | 23% | 12% | 14% | | 71% | 36% | | 16% | | 89% | | 37% | | | 9% | | 57% | | 36% | |  |
| ***10 ppm*** | 21% | 6% | 4% | | 20% | 15% | | 14% | | 86% | | 7% | | | 7% | | 31% | | 10% | |  |
| ***5 ppm*** | 14% | 1% | 3% | | 5% | 12% | | 12% | | 48% | | 2% | | | 5% | | 27% | | 4% | |  |
| ***0 ppm*** | 0% | 0% | 0% | | 0% | 0% | | 0% | | 0% | | 0% | | | 0% | | 0% | | 0% | |  |
