# Supplementary material for: Physiological and transcriptional responses of lactic acid bacteria to sub-inhibitory cadmium exposure
Source: Front Microbiol. 2026 Oct 13;17:1914052. doi: 10.3389/fmicb.2026.1914052 (PMC13712412; doi:10.3389/fmicb.2026.1914052)
Supplement: Supplementary file 4 [file Table_2.docx]

**Table S2**. Design parameters of the primers for the cadmium resistance-associated genes

| **Species** | **Gene** | **Genome start** | **Genome end** | **Forward primer (5'–3')** | **Reverse primer (5'–3')** | **GC (%)** | **Tm (°C)** | **Fragment size (bp)** |
| --- | --- | --- | --- | --- | --- | --- | --- | --- |
| ***Lm. reuteri*** | *gyrA* | 6611 | 9115 | CAATCAAAGGACGGGGTGGA | ACCAACAGCAGATCGTCCAG | 55/55 | 60.04 | 179 |
|  | *cadD* | 2097503 | 2098120 | AGCGCTTTTTGAACAGGATGAC | ACTCCAACTAAGGTTAACCACCA | 45/43 | 59.28 | 133 |
|  | *mntH* | 1781776 | 1783080 | GGGTGGCGTATTTATGGGTCTT | GAGTGATGCCACCGATTTCAG | 50/52 | 59.33 | 74 |
| ***Lt. curvatus*** | *gyrA* | 1519962 | 1522547 | TGGTCGAACGGATTGCTGAA | CACTCATATCCCGACGCACA | 50/55 | 59.97 | 120 |
|  | *cadA* | 486041 | 487834 | GGGATGAGTCGTGCTAGTCG | GCACAACTTGGTCAACCGTC | 60/55 | 59.97 | 139 |
|  | *mntH* | 890893 | 892290 | CAATCGCACAAGCACGTCAA | CCAAAGAACATTGCTGCCCC | 50/55 | 60.04 | 151 |
| ***W. confusa*** | *gyrA* | 2204617 | 2207214 | ACGTTGCCTGCATCACCTAA | TCGTGGCTCAACCTTGTCTC | 50/55 | 59.97 | 186 |
|  | *mntH* | 1462953 | 1464341 | TTCAGTTGGTACGTTTGGTCA | CGGTCAACGTACCCGTGATT | 43/55 | 60.39 | 146 |
| ***Fr. rossiae*** | *gyrA* | 6682 | 9324 | ATGCTGTTCGTGACATGGGT | GGTTTCAACACGTCAGCACC | 50/55 | 59.97 | 94 |
|  | *czcA* | 102792 | 103721 | CCATGCACCAGGAAGGTCAT | GGCTTGCTCGACCGATGATA | 55/55 | 59.97 | 138 |
|  | *cadD* | 2346928 | 2347536 | TACATCAACCGTTGTGGCGA | CCATGAGCGATAGCCTTCGT | 50/55 | 59.97 | 112 |
|  | *mntH* | 505169 | 506758 | GAGTGTCGCGTTACCGTTTG | CCGTCCACTTTACCCATGCTT | 55/52 | 60.61 | 101 |
