# Supplementary material for: Physiological and transcriptional responses of lactic acid bacteria to sub-inhibitory cadmium exposure
Source: Front Microbiol. 2026 Oct 13;17:1914052. doi: 10.3389/fmicb.2026.1914052 (PMC13712412; doi:10.3389/fmicb.2026.1914052)
Supplement: Supplementary file 5 [file Table_3.docx]

**Supplementary Table 3.** Stability assessment of the reference *gyr* gene across cadmium treatments used for RT-qPCR normalization.

| Strains | Treatment | Mean Ct ± SD |
| --- | --- | --- |
| *W.confusa* | CTRL | 16,41 ± 0,08 |
|  | 1/8 × MIC | 16,67 ± 0,04 |
|  | 1/4 × MIC | 16,02 ± 0,07 |
|  | 1/2 × MIC | 16,98 ± 0,07 |
| *Lt.curvatus* | CTRL | 18,24 ± 0,12 |
|  | 1/8 × MIC | 18,89 ± 0,08 |
|  | 1/4 × MIC | 18,36 ± 0,09 |
|  | 1/2 × MIC | 18,08 ± 0,02 |
| *Lm. reuteri* | CTRL | 19,91 ± 0,23 |
|  | 1/8 × MIC | 19,26 ± 0,18 |
|  | 1/4 × MIC | 19,77 ± 0,34 |
|  | 1/2 × MIC | 19,93 ± 0,15 |
